# The Fluoride Content of Yerba Mate Depending on the Country of Origin and the Conditions of the Infusion

**DOI:** 10.1007/s12011-015-0302-y

**Published:** 2015-03-17

**Authors:** A. Łukomska, K. Jakubczyk, D. Maciejewska, I. Baranowska-Bosiacka, K. Janda, M. Goschorska, D. Chlubek, B. Bosiacka, I. Gutowska

**Affiliations:** 1 Department of Biochemistry and Human Nutrition, Pomeranian Medical University, Broniewskiego 24 Street, 71-460 Szczecin, Poland; 2 Department of Biochemistry, Pomeranian Medical University, PowstańcówWlkp. Av. 72, 70-111 Szczecin, Poland; 3 Department of Plant Taxonomy and Phytogeography, University of Szczecin, Wąska 13 Street, Szczecin, Poland

**Keywords:** Fluoride, Yerba mate, Fluoride releasing, Temperature-depending release

## Abstract

There are many reports of the positive effect of yerba mate on the human body. Elemental composition analysis of yerba mate revealed the presence of many microelements and macroelements, but there is no literature data referencing the content and the effect of the method of preparing the yerba mate infusion on the amount of released fluoride and thus the amount of this element supplied to the human body. Therefore, in the traditional way (cold and hot), we prepared infusions of yerba mate from different countries and determined in samples content of fluoride using potentiometric method. Hot infusions resulted in statistically significant (*p* = 0.03) increases in the amount of fluoride released from the dried material to the water, compared to brewing with water at room temperature. The successive refills of hot water also resulted in a release of the same amount of fluoride, although smaller than the infusion with water at room temperature (at the third refill, it was statistically significantly smaller at *p* = 0.003). With an increase in the number of hot water refills, the amount of fluoride released from the sample portion significantly decreased. Similar results were recorded when analyzing samples depending on the country of origin. The amount of fluoride released into the water differed statistically significantly depending on the country of origin. The most fluoride was determined in the infusions of yerba mate from Argentina and the least in infusions from Paraguay.

## Introduction

Yerba mate, from the dried leaves of *Ilex paraguariensis*, is one of the most widely consumed drinks in Latin America. Its natural range includes countries such as Brazil, Argentina, Paraguay, and Uruguay. The manner and amount of consumption of yerba mate depend on regional tradition [1]. There are two general methods of mate preparation: The first is flooding dried leaves with cold water, while the second one is based on brewing with hot but not boiling water. The same dried leaves can be infused repeatedly, depending on consumer preference. In generally, 50 g of dried plant is repeatedly flooded with smaller portions of total amount of 1 L of water [1]. In these countries, 50 g of dried plant per liter of water is a daily portion [1].

There are many reports of the positive effect of yerba mate on the human body. It has been shown to lower blood cholesterol, exert a protective effect on the liver [2], and stimulate the central nervous system [3]. Some studies indicate that yerba mate helps in the prevention of cardiovascular diseases [4] and obesity [5]. Its positive effect is associated with the presence of many biologically active substances such as antioxidants [6], polyphenols, xanthine alkaloids, flavonoids, amino acids, some elements (P, Fe, Ca), and vitamins [1]. Tests on human cancer hepatoma cells (HepG2) showed the cytotoxicity of the extract of yerba mate associated with the inhibition of topoisomerase II [7]. On the other hand, some epidemiological studies indicate a relationship between the consumption of yerba mate and the presence of oral, oropharyngeal, esophageal, laryngeal, and bladder cancers [1].

Elements are essential in human nutrition, and their content in the body depends primarily on their occurrence in soil, drinking water, and food [8]. A deficiency or excess of any of the chemical elements may cause adverse effects on the human body [9, 10], especially in children [11]. There are many commercially available yerba mate blends, with different compositions and from different regions of the world. Elemental composition analysis of yerba mate revealed the presence of many microelements and macroelements [12], but there is no literature data referencing the content and the effect of the method of preparing the yerba mate infusion on the amount of released fluoride and thus the amount of this element supplied to the human body.

## Material and Methods

### Characteristics of Samples and Preparations of Yerba Mate Infusions

We analyzed 12 yerba mate tea blends. The samples were mixtures of dried leaves and stalks of different varieties of *Ilex paraguariensis*. Three samples originated in Brazil, four from Argentina, and five from Paraguay. Ten gram samples from each mixture were taken for analysis.

#### Cold Infusion

Ten gram test portions were placed in plastic cups. Fifty milliliter of water at room temperature (25 °C) was poured over the samples and allowed to stand for 10 min. After that time, the infusions were filtered into 50 mL tubes.

#### Hot Infusion

Ten gram test portions were placed in plastic cups; 50 mL of water at 85 °C was poured over the tea and left to stand for 10 min. After that time, the infusions were filtered into 50 mL tubes.

The procedures were repeated two more times, flooding the same yerba mate tea samples. Infusions from each filtration were placed in separate tubes.

### Determination of Fluoride Content in Prepared Samples

Sample levels of F^−^ were determined using a potentiometric ion-selective electrode (Thermo Scientific Orion, USA) according to the works of Gutowska et al. [13, 14]. The fluoride content in samples was calculated based on the difference of potentials measured in each sample and the concentration of the added standard. The electrode had been calibrated using standard solutions.

### Statistic Analysis

Statistical analysis was performed using Stat Soft Statistica 10.0 and Microsoft Excel 2007. The arithmetic means (AMs) and standard deviations of the AM (SDs) were calculated for each studied group. The distribution of results for individual variables was obtained with the Shapiro–Wilk W test. As most of the distributions deviated from the normal Gaussian distribution, non-parametric tests were used for further analyses. To assess the differences between the studied groups, the non-parametric Mann–Whitney test was used. The level of significance was *p* ≤ 0.05.

## Results

**Fig. 1:**
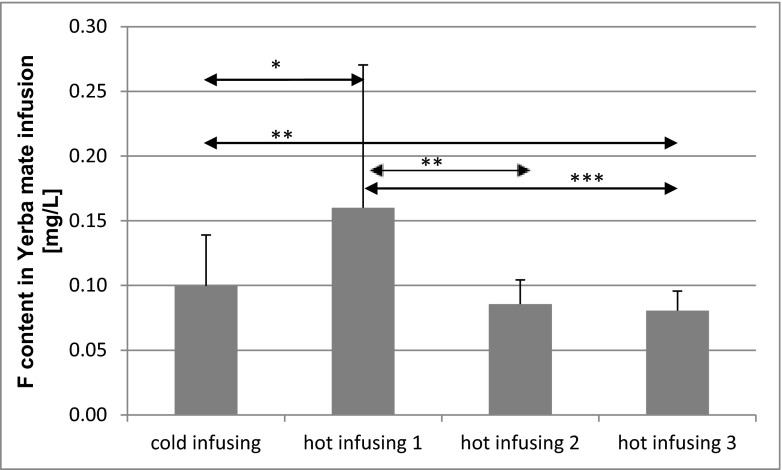
Fluoride content in yerba mate infusion in dependence on conditions for brewing the tea probes from Brazil, Argentina, and Paraguay. *p < 0.05, **p < 0.01, ***p < 0.001—statistically significant differences

The fluoride concentration in the yerba mate infusions changed depending on the conditions of brewing. Hot infusions resulted in statistically significant (*p* = 0.03) increases in the amount of fluoride released from the dried material to the water, compared to brewing with water at room temperature. The successive refills of hot water also resulted in a release of the same amount of fluoride, although smaller than the infusion with water at room temperature (at the third refill, it was statistically significantly smaller at *p* = 0.003). With an increase in the number of hot water refills, the amount of fluoride released from the sample portion significantly decreased (Fig. 1).

**Fig. 2:**
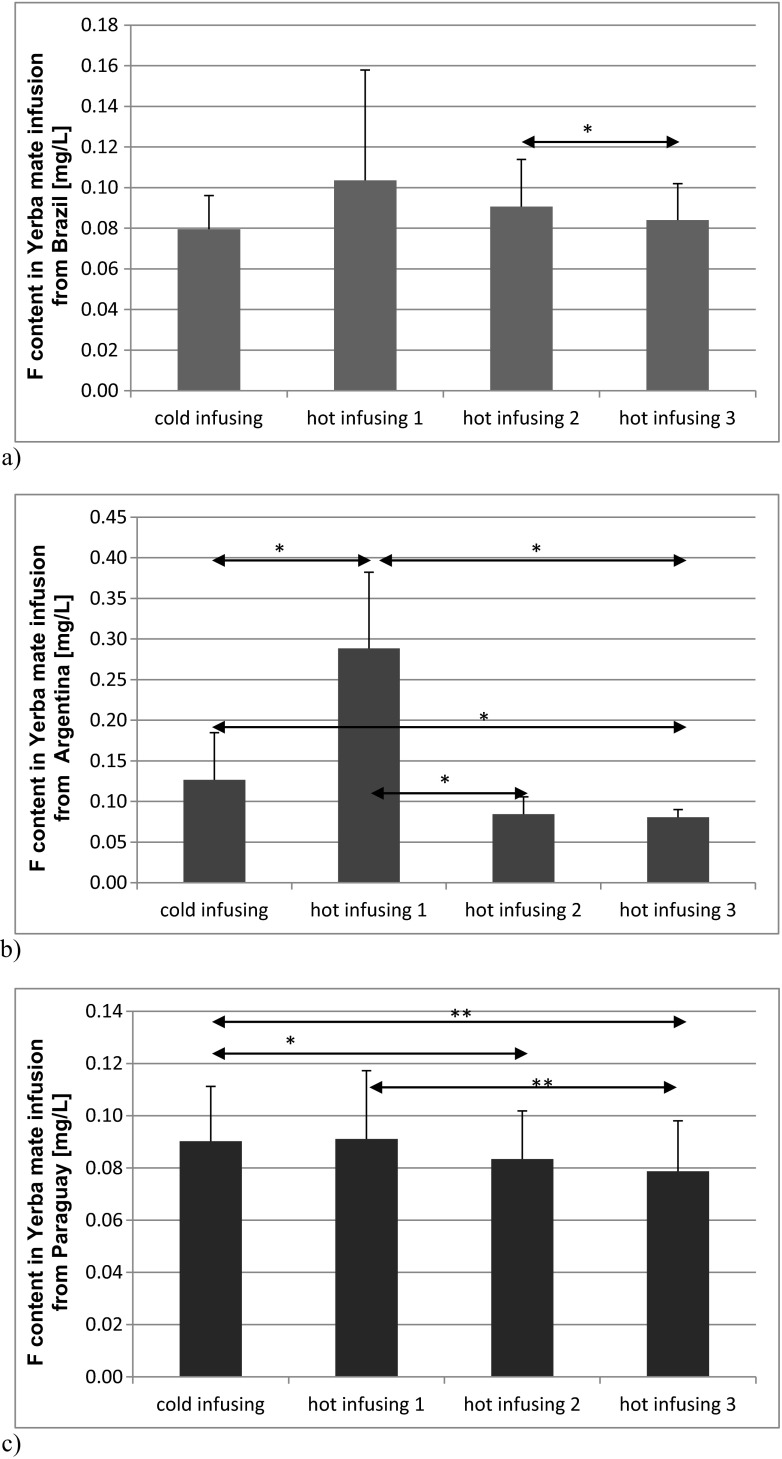
Fluoride content in yerba mate infusion in dependence on conditions for brewing the tea probes and origin of the samples: **a** Brasil, **b** Argentina, **c** Paraguay. *p < 0.05, **p < 0.01, ***p < 0.001—statistically significant differences

Similar results were recorded when analyzing samples depending on the country of origin. The amount of fluoride released into the water differed statistically significantly depending on the country of origin. The most fluoride was determined in the infusions of yerba mate from Argentina (cold and hot infusion 1) and the least in infusions from Paraguay (hot infusions 1, 2, and 3) and Brazil (cold infusion) (Fig. 2).

## Discussion

It is well known that in people, macronutrients such as calcium and magnesium are needed in large quantities, while trace elements, including fluorine, are needed in low concentrations for several physiological functions [15]. Fluorine is one of the trace elements with an important biological role [16]. It is found in all body tissues, but its greatest accumulation occurs in bones and teeth [13, 17], where it is part of fluoroapatite responsible for the hardness and strength of the bone [18] and the solubility of tooth enamel [14].

There are certain food products believed to have properties that protect teeth from the occurrence and development of caries. These include milk, cheese, cocoa beans, unprocessed plant foods, and tea [19], which has been studied in detail because of its ability to accumulate large amounts of fluoride from the soil [20, 21].

Research in this study showed a difference in the amount of fluoride between the infusions of yerba mate from different countries, possibly due to differences in the quality of the soil, similar to black tea (*Camellia sinensis*). For most plants, fluorine absorbed from a soil rich in F is phytotoxic, but tea has the ability to absorb and accumulate massive amounts of fluoride in mature leaves without exhibiting toxic symptoms, even when grown in areas with a low content of fluorine in the soil [20]. These observations have been confirmed in studies of other authors, which showed that young tea leaves contain less fluoride than old leaves [21]. Research conducted by Stevens et al. [22] shows that the amount of fluoride ions absorbed by the plant largely depends on the chemical form in which this element is present in the soil. Because HF is more soluble in the aqueous environment, it can be quickly taken up by the plant; in addition, tea bushes prefer to grow on soils with acidic pH, which facilitates the withdrawal of fluorine [22]. Moreover, the differences in technological processes used in the production of tea affect its quality [23] and fluoride content in the dried leaves and infusions [24].

Fluoride levels were highest in samples from Argentina. This is most likely due to the geographical location of Argentina near the seismically active region of the Ring of Fire. As reported by the study of Francisca et al. [25], the level of fluoride in groundwater in Argentina is elevated and ranges between 0 and 13.5 mg/L, with a mean of 2.36 mg/L. Note that the mean value of fluoride concentration in groundwater exceeds the limit suggested by the WHO (1.5 mg/L) [25]. After the eruption of the volcano Puyehue-Cordon The Caulle (PCCVE, June 2011) in Chile and Argentina, it has shown an increase of developing fluorosis, which seems to confirm the negative impact of the eruption on the fluorine content in the environment [26]. Probably, toxic fluorine-containing compounds that are released during volcanic eruptions, have a strong impact on the rise amount of fluoride in yerba mate teas originating in Argentina compared to teas from Brazil and Paraguay.

For people, tea is one of the essential dietary sources of fluorine. It contains relatively large amounts of this element, which are released during brewing [27]. The absorption of fluoride from tea infusions with “normal” levels of this element are considered to be safe and may provide a way to protect teeth from the development of caries [28]. However, several studies have shown that very high daily consumption of tea or drinking certain types of tea, for example, “brick tea”, which contain very high concentrations of fluoride, may lead to the development of fluorosis [28, 29].

Malinowska et al. [24] tested commercially available teas: black, green, white, oolong and pu-erh, in terms of the release of fluoride to the water depending on the length of brewing. It was noted that with an increasing length of brewing, fluoride content in the infusion also increased. Moreover, the infusion of black tea had the highest concentration of this element (10 times higher) than the other types of tea. The range of results ranged from 4.54 mg/L for black tea to 0.54 mg/L for white tea, and 0.09 mg/L for herbal infusions. The amount of fluoride absorbed into the body depends on the number of cups of tea consumed per day and assuming a daily intake is five cups (1 L of tea per day), the daily supply of fluoride in the body can range from 8 % for herbal teas to more than 303 % for black teas compared to the Polish standard safe and adequate daily intake (SAI) and acceptable daily intake (ADI) [24] (respectively, from 0.9 to 3 % for herbal teas to 151.3 % for black teas compared to the US standards determined by National Academy of Sciences, Food and Nutrition Board, USA), which may be a cause of excessive accumulation of this element in the hard tissues of the body [13, 28, 29] and the exposure of the whole system to its negative effects by increasing the synthesis of ROS in cells [30]. Drinking five cups of yerba mate per day, we delivered to the body between 0.08 and 0.29 mg of fluoride.

The US recommended adequate intake (AI) levels for fluoride are of 3–4 mg/day, and a upper intake level (UL) is of 10 mg/day for adults (Dietary Reference Intakes recommended by the National Academy of Sciences, Food and Nutrition Board, USA) [31]; in Poland, RDA for fluoride is 3–4 mg per day for adults (Food and Nutrition Institute in Warsaw). However, the recommended daily intake of fluoride is variable depending on gender and age [24]. This amount satisfies from 2 to 7.25 % of the daily intake of fluorine for adults according to standards of Food and Nutrition Institute in Warsaw (respectively, 1–3.5 % according to standards of National Academy of Sciences, Food and Nutrition Board, USA). Potential toxic dose (PTD) of fluoride has been set at 5 mg F/kg body weight [32].

Due to the specific nature of the brewing process in yerba mate, namely several infusions from the same leaves or brewing in water at room temperature, single portions provide much lower average amounts of fluoride than black tea, in which a new batch of dried leaves is infused with hot water each time. However, even this low amount, when intaken on a regular basis, it may have a significant effect on the body. In a study on postmenopausal women by Conforti et al. [33], bone mineral density at the spine and hip was greater in the case of the group regularly drinking yerba mate infusions compared to the group that did not. The increase in the accumulation of fluoride in hard tissues can interrupt the apatite crystal nucleation process and result in crystal defects [34], which may reduce bone quality despite the increase in bone mass [35].

People are often exposed to fluorine compounds from a variety of sources, such as food, water, air, and the excessive use of toothpaste and products for oral hygiene containing fluorine compounds. Tea, one of the most popular beverages, may also be one of those sources. An excessive intake of fluoride from black tea, especially in areas with high levels of this element in the water, may increase the risk of dental fluorosis, especially in children who are developing permanent teeth. In addition, long-term exposure to high doses of fluoride can lead to bone fluorosis [24].

## References

[1] Heck CI, Mejia EG (2007). Yerba mate Tea (*Ilex pagaguariensis*): a comprehensive review on chemistry, health implications, and technological considerations. J Food Sci.

[2] Filip R, Ferrano GE (2003). Researching on new species of “mate”: *Ilex breicuspis*: phytochemical and pharmacology study. Eur J Nutr.

[3] Gonzalez A, Ferreira F, Azguez A, Moyna P, Paz EA (1993). Biological screening of Uruguayan medicinal plants. J Ethnopharmacol.

[4] Schinella G, Fantinelli JC, Mosca SM (2005). Cardioprotective effects of *Ilex paraguariensis* extract: evidence for a nitric oxide-dependent mechanism. Clin Nutr.

[5] Andersen T, Fogh J (2001). Weight loss and delayed gastric emptying following a South American herbal preparation in overweight patients. J Hum Nutr Diet.

[6] Filip R, Lotito SB, Ferraro G, Fraga CG (2000). Antioxidant of *Ilex paraguariensis* and related species. Nutr Res.

[7] Ramirez-Mares MV, Chandra S, de Mejia EG (2004). In vitro chemopreventive activity of camellia sinensis, *Ilex paraguariensis* and ardisiacompressa tea extracts and selected polyphenols. Mutat Res.

[8] Panczenko-Kresowska B, Ziemlański Ś, eds (2001) Mineral elements—their importance inhuman nutrition. Standards of human nutrition. Physiologicalbasis. PZWL, Warszawa, Poland, 309–410

[9] Graczyk A (2010). Essential and toxic—the role of the elements in the body. World Pharm.

[10] Pendrys D (2000). Risk of enamel fluorosis in nonfluoridated populations: consideration for the dental professional. J Am Dent Assoc.

[11] Stencel-Gabriel K, Lukas A (2006). The maturation of the immune system of the newborn. Prob Family Med.

[12] Bragança VL, Melnikov P, Zanoni LZ (2011). Trace elements in different brands of yerba mate tea. Biol Trace Elem Res.

[13] Gutowska I, Baranowska-Bosiacka I, Szynkowska A, Siwiec E, Szczuko M, Noceń I, Rębacz-Maron E, Chlubek D, Stachowska E (2012). Effect of supplementation with conjugated dienes of linoleic acid (CLA) on the content of fluoride, calcium and magnesium in the hard tissues and serum of mice. Fluoride.

[14] Gutowska I, Baranowska-Bosiacka I, Noceń I, Dudzińska W, Marchlewicz M, Wiszniewska B, Chlubek D (2011). Changes in the concentration of elements in the teeth of rats with type 1 diabetes, in the peak stage of the disease with absolute insulin deficit. Biol. Trace Elem. Res.

[15] Apostoli P (2002). Element in environmental and occupational medicine. J. Chromatogr. B Anal. Technol. Biomed. Life Sci.

[16] Dholam KP, Somani PP, Prabhu SD, Ambre SR (2013). Effectiveness of fluoride varnish application as cariostatic and desensitizing agent in irradiated head and neck cancer patients. Int J Dent.

[17] Chachra D, Limeback H, Willett TL, Grynpas MD (2010). The long-term effects of water fluoridation on the human skeleton. J Dent Res.

[18] Fernandes MD, Yanai MM, Martins GM, Iano FG, Leite AL, Cestari TM, Taga R, Buzalaf MA, de Oliveira RC (2014). Effects of fluoride in bone repair: an evaluation of RANKL, OPG and TRAP expression. Odontology.

[19] Moynihan P (2000). Foods and factors that protect against dental caries. Nutr Bull.

[20] Xie Z, Ye ZH, Wong MH (2001). Distribution characteristics of fluoride and aluminium in soil profiles of an abandoned tea plantation and their uptake by six woody species. Environ Int.

[21] Ruan J, Wong MH (2001). Accumulation of fluoride and aluminium related to different varieties of tea plant. Environ Geochem Health.

[22] Stevens DP, McLaughlin MJ, Alston AM (1998). Phytotoxicity of hydrogen fluoride and fluoroborate and their uptake from solution culture by lycopersiconesculentum and Avena sativa. Plant Soil.

[23] Sinija VR, Mishra HN, Bal S (2007). Process technology for production of soluble tea powder. J Food Eng.

[24] Malinowska E, Inkielewicz I, Czarnowski W, Szefer P (2008). Assessment of fluoride concentration and daily intake by human from tea and herbal infusions. Food Chem. Toxicol.

[25] Francisca FM, Carro Perez ME (2009). Assessment of natural arsenic in groundwater in Cordoba Province, Argentina. Environ Geochem Health.

[26] Flueck WT, Smith-Flueck AM (2013). Severe dental fluorosis in juvenile deer linked to a recent volcanic eruption in Patagonia. J. Wildl. Dis.

[27] Rao GS (1984). Dietary intake and bioavailability of fluoride. Annu. Rev. Nutr.

[28] Simpson A, Shaw L, Smith AJ (2001). The bio-availability of fluoride from black tea. J Dent.

[29] Fung KF, Zhang ZQ, Wong JWC, Wong MH (1999). Fluoride contents in tea and soil from tea plantations and the release of fluoride into tea liquor during infusion. Environ. Pollut.

[30] Gutowska I, Baranowska-Bosiacka I, Baśkiewicz M, Millo B, Siennicka A, Marchlewicz M, Wiszniewska B, Machaliński B, Stachowska E (2010). Fluoride as a pro-inflammatory factor and inhibitor of ATP bioavailability in differentiated human THP1 monocytic cells. Toxicol. Lett.

[31] Food and Nutrition Board, Institute of Medicine (1997). Fluoride dietary reference intakes for calcium, phosphorus, magnesium, vitamin D, and fluoride.

[32] Whitford GM (2011). Acute toxicity of ingested fluoride. Fluoride and the oral environment. Monogr Oral Sci Basel, Karger.

[33] Conforti AS, Gallo ME, Saraví FD (2012). Yerba mate (Ilex paraguariensis) consumption is associated with higher bone mineral density in postmenopausal women. Bone.

[34] Kakei M, Sakae T, Yoshikawa M, Tamura N (2007). Effect of fluoride ions on apatite crystal formation in rat hard tissues. Ann Anat.

[35] Aaron JE, de Vernejoul MC, Kanis JA (1991). The effect of sodium fluoride on trabecular architecture. Bone.

